# An Experience of Donor Lymphocyte Infusion after Reduced-Intensity Conditioning Allogeneic Hematopoietic Stem Cell Transplantation for Chronic Granulomatous Disease

**DOI:** 10.3390/pediatric12030030

**Published:** 2020-12-11

**Authors:** Tahereh Rostami, Azadeh Kiumarsi

**Affiliations:** Hematology-Oncology and Stem Cell Transplantation Research Institute, Shariati Hospital, Tehran 1411713131, Iran; trostami@sina.tums.ac.ir

**Keywords:** donor lymphocyte infusion, reduced-intensity conditioning, allogeneic hematopoietic stem cell transplantation, chronic granulomatous disease, CGD

## Abstract

Allogeneic hematopoietic stem-cell transplantation is a well-known curative treatment for patients with chronic granulomatous disease. We present our experiment regarding ten patients with chronic granulomatous disease who underwent a reduced intensity conditioning regimen consisting of melfalan, fludarabine, and antithymocyte globulin. Donor lymphocyte infusion was used in three representative patients who developed mixed donor chimerism. After at least 2 years of median follow-up, 8 of the 10 patients are alive and well.

## 1. Introduction

Hematopoietic stem cell transplantation (HSCT) is the only curative treatment for patients with Chronic granulomatous disease (CGD) which is a primary immunodeficiency disorder characterized by an inherited defect in one of five subunits of the neutrophilic NADPH oxidase that leads to life threatening bacterial and fungal infections. In addition to the proneness for infection, hyperinflammation and noninfectious inflammatory complications such as colitis are important parts of the symptomatology in CGD [1,2]. Myeloablative conditioning regimens have been historically employed in CGD, but due to myeloablative several disadvantages including greater susceptibility to tissue injury, longer duration of neutropenia, and fears of secondary tumors, nowadays, reduced-intensity conditioning (RIC) regimens are being used progressively more in different centers [3,4,5,6]. RIC regimens have been used to diminish transplant-related mortality, but carry the risk of mixed donor-recipient chimerism that may progress to graft loss [7]. The likelihood of graft rejection in the mentioned approach could be reduced by infusions of donor lymphocytes after transplantation [8,9,10].

We describe here the experience of our center with a reduced toxicity regimen for patients with CGD and donor lymphocyte infusion (DLI) in those who appeared to have mixed chimerism.

## 2. Methods

We conducted a retrospective analysis of ten patients who underwent allogeneic HSCT for CGD at Shariati hospital, Hematopoietic Stem Cell Transplant Research Center (HORCSCT), Tehran, Iran. The characteristics of the patients are listed in Table 1. The diagnosis of CGD in our patients was confirmed by nitroblue tetrazolium test (NBT) [11]. Unfortunately, molecular diagnosis and genetic testing was not available for any of the patients. Human leukocyte antigen (HLA) matching of the donors and recipients was confirmed by molecular typing of the HLA class I and HLA class II loci and all the donors were fully matched. All the patients were using sulphamethoxazole-trimethoprim and itraconazole as bacterial and fungal infection prophylaxis at the time of stem-cell transplantation. Four of the patients were receiving interferon gamma therapy at the time of HSCT; five of the patients were on antituberculosis prophylaxis by isoniazid due to history of disseminated BCGosis or pulmonary tuberculosis.

All patients had undergone a conditioning regimen consisting of melphalan at a dose of 70 mg per square meter of body-surface area (days 7 and 6 before transplantation), fludarabine at a dose of 30 mg per square meter of body-surface area (days 5 to 1 before transplantation), and antithymocyte globulin at a dose of 2.5 mg per kilogram (days 5 to 2 before transplantation). Administration of cyclosporine was started on day 4 before transplantation.

## 3. Results

From May 2012 to October 2016, ten patients with CGD were submitted to HSCT in our center. Median age at transplant was 9 years (range 1–15).

The least follow-up time was 2 years. The median duration of neutropenia (defined as an absolute neutrophil count of <500 per cubic millimeter) was 11 days (range, 8 to 11), and the median duration of thrombocytopenia (defined as a platelet count of <20,000 per cubic millimeter) was 13 days (range: 11 to 15). Characteristics of HSCT course in the patients are summarized in Table 2.

Acute graft versus host disease (GVHD) grade II-III was encountered in 4 patients (1 = only skin and 1 = skin, gut and liver, 1 = only gut and 1 = only liver). One patient developed limited chronic pulmonary GVHD.

**Figure 1:**
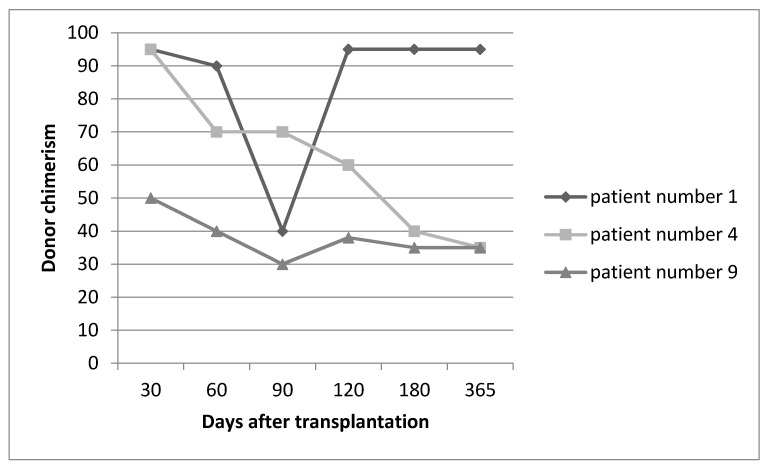
Sequence of donor chimerism in three representative patients who underwent DLI.

Three month after HSCT, low donor chimerism (less than 40%) was encountered in 4 patients, among which DLI was employed for 3 patients. In the first patient, after one episode of DLI, chimerism was reached from 40% to 95%. In two other patients, even after three episodes of DLI, donor chimerism was sustained at a level of about 30% with which patients remained symptom free until the present time (Figure 1). In one of the patients with mixed chimerism due to unavailability of the donor’s vein access, DLI could not happen; however, with only tapering the dose of cyclosporine, donor chimerism reached 95% within 2 months.

As a final point, eight of ten patients are alive and symptom free at the present time.

## 4. Discussion

Allogeneic HSCT with reduced toxicity regimen is a good alternative in the treatment of patients with CGD. However, it carries an increased risk of incomplete donor hematopoietic cells engraftment or graft rejection. Studies have shown that even partial engraftment of donor stem cells is able to restore normal immune function in patients with CGD [12]. Donor lymphocytes infusion could be an aid for overcoming incomplete engraftment in these patients. Horwitz et al. using a nonmyeloablative T cell depleted HSCT in patients with CGD, confirmed the existing data showing that infusion of donor lymphocytes in graded increments could facilitate engraftment of donor stem cells [5,10]. In our experiment, DLI helped one of the patients to reach full donor chimerism and helped the other two to stay symptom free.

Considering the transplant related mortality, our only patient whose donor was unrelated, died due to sepsis, and one of our patients developed deterioration of mental status and progressive encephalopathy after receiving fludarabine, which is a rare but known adverse effect of the mentioned drug [13,14]. She unfortunately died due to complications of CNS toxicity while developing full donor chimerism. Other series have reported death due to progressive fungal infection and severe GvHD in their patients with CGD after HSCT [3].

From the GvHD point of view, none of our patients encountered severe GvHD. In a survey of the European experience, using a myeloablative busulphan-based regimen, severe GVHD developed in 4 out of 27 patients [13]. Interestingly, those patients who had mixed donor chimerism did not develop GvHD. It is reported that mixed hematopoietic chimerism is able to suppress GvHD occurrence with poorly understood mechanisms [15,16,17,18].

In conclusion, we suggest that the HSCT with a reduced intensity conditioning regimen from an HLA-identical sibling could benefit patients with CGD, and DLI could be useful for those who are leaning towards graft failure.

## Figures and Tables

**Table 1:** Characteristics of the patients and donors.

Patient Number	Age at HSCT (Year)	Sex	Donor Type	Donor Age (Year)	Stem Cell Source *	CD3+ Cells × 10^6^ per kilogram	CD34+ Cells × 10^6^ per kilogram
1	13	m	sibling	28	PB	430	1.4
2	7	f	unrelated	N/A	PB	180	2.2
3	15	m	sibling	8	PB	270	6.7
4	3.5	m	sibling	3.5	PB	155	3.5
5	7.5	f	mother	35	BM	74	2.4
6	11	f	sibling	16	PB	211	6.2
7	15	f	sibling	29	PB	255	7.45
8	2	f	sibling	3	PB	74	0.3
9	12	f	sibling	10	PB	348	3.45
10	1	f	sibling	5	PB	191	5.77

* PB = Peripheral blood, BM = Bone marrow.

**Table 2:** Characteristics of HSCT course in the patients.

Patient Number	Duration of Neutopenia *	Duration of Thrombocytopenia †	Number of Red Cell Transfusion Required	NBT 30 Days after HSCT	NBT 2 Years after HSCT	Donor Chimerism 30 Days after HSCT	Donor Chimerism 2 Years after HSCT	Transplant Related Mortality	Graft Versus Host Disease	DLI
1	11	14	1	90	100	95	95	No	Liver and lung	yes
2	11	died	6	30	-	5	-	Yes, due to sepsis	no	-
3	11	11	1	100	100	95	95	No	no	-
4	11	12	3	95	72	95	95	No	no	yes
5	11	13	1	100	90	95	95	No	Gut, liver and skin	-
6	8	died	several	100	-	95	-	Yes, due to drug adverse effect	Gut	-
7	9	14	3	100	100	95	95	No	Skin	-
8	9	11	2	100	100	95	95	No	no	-
9	11	13	1	20	35	50	38	No	no	yes
10	11	15	3	100	99	95	95	No	no	-

* Neutropenia was defined as an absolute neutrophil count of less than 500/mL^3^. † Thrombocytopenia was defined as a platelet count of less than 20,000/mL^3^.
